# A novel swine model for evaluation of dyslipidemia and atherosclerosis induced by human *CETP* overexpression

**DOI:** 10.1186/s12944-017-0563-x

**Published:** 2017-09-11

**Authors:** Tao Chen, Meng Sun, Jia-Qiang Wang, Jin-Jin Cui, Zhong-Hua Liu, Bo Yu

**Affiliations:** 1 The Key Laboratory of Myocardial Ischemia, Chinese Ministry of Education, Harbin, Heilongjiang China; 2 Cardiology Division, The Second Affiliated Hospital of Harbin Medical University, No. 246 Xuefu Road, Harbin, Heilongjiang 150086 China; 3 College of life science, Northeast Agricultural University of China, Harbin, China

**Keywords:** Cholesteryl ester transfer protein, Transgenic pig, Atherosclerosis, Metabolism

## Abstract

**Background:**

The mechanism of cholesteryl ester transfer protein (CETP) in lipid metabolism is still unclear. Furthermore, the relationship of CETP and atherosclerosis (AS) has been controversial. As pigs are a good model for both lipid and AS research, we investigated the lipid metabolism of human *CETP* (*hCETP*) transgenic pigs and explored the mechanism of CETP in lipid modulation.

**Methods:**

Plasmids expressing the *hCETP* gene were designed, successfully constructed, and transfected into porcine fetal fibroblasts by liposomes. Using somatic cell nuclear transfer technology and embryonic transfer, *hCETP* transgenic pigs were generated. After the DNA, RNA, and protein levels were identified, positive *hCETP* transgenic pigs were selected. Blood samples were collected at different ages to evaluate the phenotypes of biochemical markers, and the metabolomes of plasma samples were analyzed by liquid mass spectrometry.

**Results:**

Eight positive *hCETP* transgenic pigs and five negative cloned pigs were generated by transgenic technology. Finally, five *hCETP* transgenic and five cloned pigs were grown healthily. After feeding with a normal diet, *hCETP* transgenic pigs compared with unmodified pigs had no significant differences in body weight, liver function, kidney function, or plasma ions, while total cholesterol and low-density lipoprotein were higher than in unmodified pigs, and high-density lipoprotein was significantly decreased. Metabolomics analysis showed that there were differences in metabolic components between *hCETP* transgenic pigs, cloned pigs, and unmodified pigs.

**Conclusions:**

In this study, we created *hCETP* transgenic pigs that could serve as an excellent model for lipid disorders and atherosclerosis.

**Electronic supplementary material:**

The online version of this article (10.1186/s12944-017-0563-x) contains supplementary material, which is available to authorized users.

## Background

Cholesteryl ester transfer protein (CETP) is a 74-KDa hydrophobic glycoprotein that is secreted mainly from the liver and circulates in plasma. It is present in humans, rabbits, hamsters, chickens, and primates, but absent in rodents, pigs, cows, dogs, and horses [1]. CETP plays a critical role in lipid metabolism in humans, especially in mediating the exchange of cholesteryl esters (CE) and triglycerides (TG) between apoB-containing lipoproteins and high-density lipoprotein (HDL) [2]. The overall role of CETP in atherosclerosis is complex, and whether CETP is an antiatherogenic or proatherogenic protein has been debated for many years. The results of introducing the *CETP* gene into rodents, which are naturally *CETP*-deficient, showed reduced HDL levels and severe atherosclerosis (AS) development [3–6]. Rabbits are highly susceptible to developing diet-induced AS because of their naturally high levels of CETP. In addition, it was reported that inhibiting CETP in a rabbit model of AS results in a marked reduction in AS [7]. Furthermore, genetic variants of CETP in humans with low CETP activity may be protective against cardio-vascular disease (CVD), since they exhibit higher HDL levels and reduced low-density lipoprotein (LDL) levels [8]. CETP inhibitors have been a hot topic of CVD research in recent years. To date, several CETP inhibitors have been brought to clinical trials. Although some CETP inhibitors have proven disappointing, some have been shown to induce significant changes in lipid profiles and metabolism, which permits some optimism for their role in cardiovascular risk reduction [9]. Therefore, more clinical and experimental data is needed to fully understand CETP and substances that affect it.

The majority of animal models used in research are rodents and rabbits whose physiological systems are significantly different from humans. Moreover, the small size of these animals precludes research in intravascular devices and is a challenge for noninvasive imaging. Many important progressive features of human atherosclerosis, such as plaque angiogenesis, plaque ruptures, and thrombosis, are rare or absent in rodent models [10]. Therefore, in recent years, transgenic pigs have been considered as a promising model for scientific research with the sequencing and annotation of the pig genome, given that their genome organization, anatomy, physiology, pathology, body weight, and lifespan closely resemble those of humans [11]. Because studies looking at the effects of CETP expression on lipid metabolism and atherosclerosis have produced contradictory results, generating a new transgenic pig model will be helpful for further CETP-related research.

In the present paper, we created such a genetic model by liver- and intestine-specific overexpression of the *hCETP* gene in pigs. As the pigs aged, this model developed increased plasma total cholesterol (TC) and LDL levels, and decreased plasma triglyceride (TG) and HDL levels. Metabolomics showed some key substances were associated with CETP function in lipid metabolism.

## Methods

### Ethics statement

All animal care and experiments in this research followed the guidelines of the Second Affiliated Hospital of Harbin Medical University and were approved by the Animal Use and Care committee. All animals (pigs) involved in this research were raised and bred following the guidelines of the Animal Husbandry Department of Heilongjiang, P.R. China.

### Genetic constructs

The plasmid pEGFP-C1 (Clontech, CA, USA) was used as a skeleton. We directly subcloned human *CETP*, a 1.48-kb fragment with *NheI/BamHI* sites, from cDNA purchased from Sino Biological Inc. China (Catalog Number: HG13276-G). A 1.4-kb fragment of the human ApoC3 promoter was cloned from human genomic DNA with *AseI/NheI* sites as previously described [6]. The liver-specific expression promoter to drive the targeted *hCETP* gene and internal ribosome entry site (*IRES*) were inserted downstream of *hCETP*, followed by enhanced green fluorescence protein (*EGFP*) and an SV40 poly-A tail. Finally, the pApoCIII-hCETP-IRES-EGFP-SV40polyA gene construct was obtained whose structure is shown in Additional file 1: Figure S1-A. Primers used in PCR were as follows: human ApoC3 (Forward: 5′-ATTAATATTCTGAGGGCAGAGCCG-3′; Reverse: 5′- GCTAGCCAGCTGCCTCTAGGG-3′); *hCETP* (Forward: 5′- GCTAGCATGCTGGCTGCCAC-3′; Reverse: 5′- GGATCCCTAGCTCAAGCTCTGGAG-3′).

### Generation of hCETP transgenic pigs

Fibroblast cells derived from E32 fetuses were transfected by the liposome-mediated plasmid pApoC3-hCETP-IRES-EGFP-SV40polyA, which was based on random insertion of nonhomologous DNA vector into the host genome. After G418 selection, surviving cells were propagated and confirmed by PCR as shown in Additional file 1: Figure S1-B; these were used as nuclear donors, and nuclear transfer was performed as previously described [12]. The reconstructed oocytes were activated and cultured for 18–22 h, and the ones in a good growth state were surgically transferred into an oviduct of the surrogate. The surrogates were kept in a conventional environment for housing pigs. Pregnancies were confirmed by ultrasonography on day 28, and all of the transgenic piglets were delivered by vaginal birth 24 h after induction with prostaglandin.

### Identification of hCETP transgenic pigs

#### DNA analysis

Each DNA sample was cleaved with *EcoRI* and *NheI* (TaKaRa, Dalian, China), which can digest the pig genome efficiently. Identification of *hCETP* transgenic pigs in DNA level was done using primers by PCR. The sequences of the primers were 5′-GAGCAAGGGCGAGGAGCTGTTCA-3′ (forward) and 5′ -TGCAGAATTCGAAGCTTGAGC-3′ (reverse).

#### Real-time PCR and western bolt analysis

To examine the expression of *hCETP* in transgenic pig tissue, total RNA was isolated from 12 different tissues in two piglets. All samples were processed in triplicate, and the relative expression was standardized to GAPDH. Primers for the *hCETP* gene were 5′- CCTGACTGCTACCTGTCTTTCCA-3′ (forward) and 5′- TCCCTTCAGGACCAGCTTCAG-3′ (reverse). Total proteins were isolated from plasma of pigs No. 1, 2, 3, 4, 5, 8, 10, 11, 12, 13 for detecting CETP expression. The detailed procedures of RT-PCR and western blotting were performed as previously described [12].

### Animals and diets

The pigs were weaned at 28 days and fed a standard diet. All big white pigs were obtained and housed at the Northeast Agriculture University Research Institute (Harbin, China). Pigs had access to autoclaved water and normal diet (Animal husbandry of YuanDa, Harbin, China) ad libitum. Body weight was measured every month.

### Plasma analysis

Blood samples were drawn from jugular veins into EDTA-coated tubes after an overnight fast at the time points indicated. Then they were stored on ice and centrifuged within 1.5 h at 1800 rpm for 10 min at 4 °C. All samples were stored at −80 °C until analysis. Plasma TC, HDL, LDL, TG, glucose, liver function, and renal function were measured according to standard laboratory procedures of the Second Affiliated Hospital of Harbin Medical University.

### Metabolic analysis

#### Sample preparation

Before RRLC-QTOF/MS analysis, the plasma and QC samples were thawed and refrozen in a 4 °C water bath. A volume of 1500 mL of methanol was added to 300 mL of plasma. After vortexing vigorously for 2 min, the mixture was allowed to settle at 20 °C for 10 min, and then centrifuged at 14,000 *g* for 15 min at 4 °C. The supernatant was transferred to a clean vial and dried under nitrogen at 37 °C. The residue was dissolved in 300 mL of acetonitrile–water (3:1, *v*/v), kept at 30 °C for 10 min, and then vortex mixed for 60 s. The supernatant was then placed into the sample vial for RRLC-QTOF/MS analysis [13].

#### Liquid chromatography mass spectrometry

A 10 mL aliquot of the pre-treated sample was injected into a 3.0100 mm (1.8 mm) ZORBAX SB-C18 column (Agilent Technologies, Santa Clara, CA, USA) using a rapid resolution liquid chromatography system (1260 series, Agilent Technologies). All samples were maintained at 4 °C during the analysis [14]. Mass spectrometry was performed on an Agilent 6530-QTOF (Agilent Technologies) equipped with an electrospray ionization source operating in electrospray positive (ESI^+^) and electrospray negative (ESI^−^) modes. The detailed methods were as previously described [15].

#### Statistical analysis

The experimental data of each group were expressed as means ± standard deviation. Student’s *t-*test was applied to determine the differences between groups, and statistical significance was set at *P* < 0.05. Statistical analysis was performed using SPSS13.0 software.

## Results

### Production of hCETP transgenic pigs

**Fig. 1:**
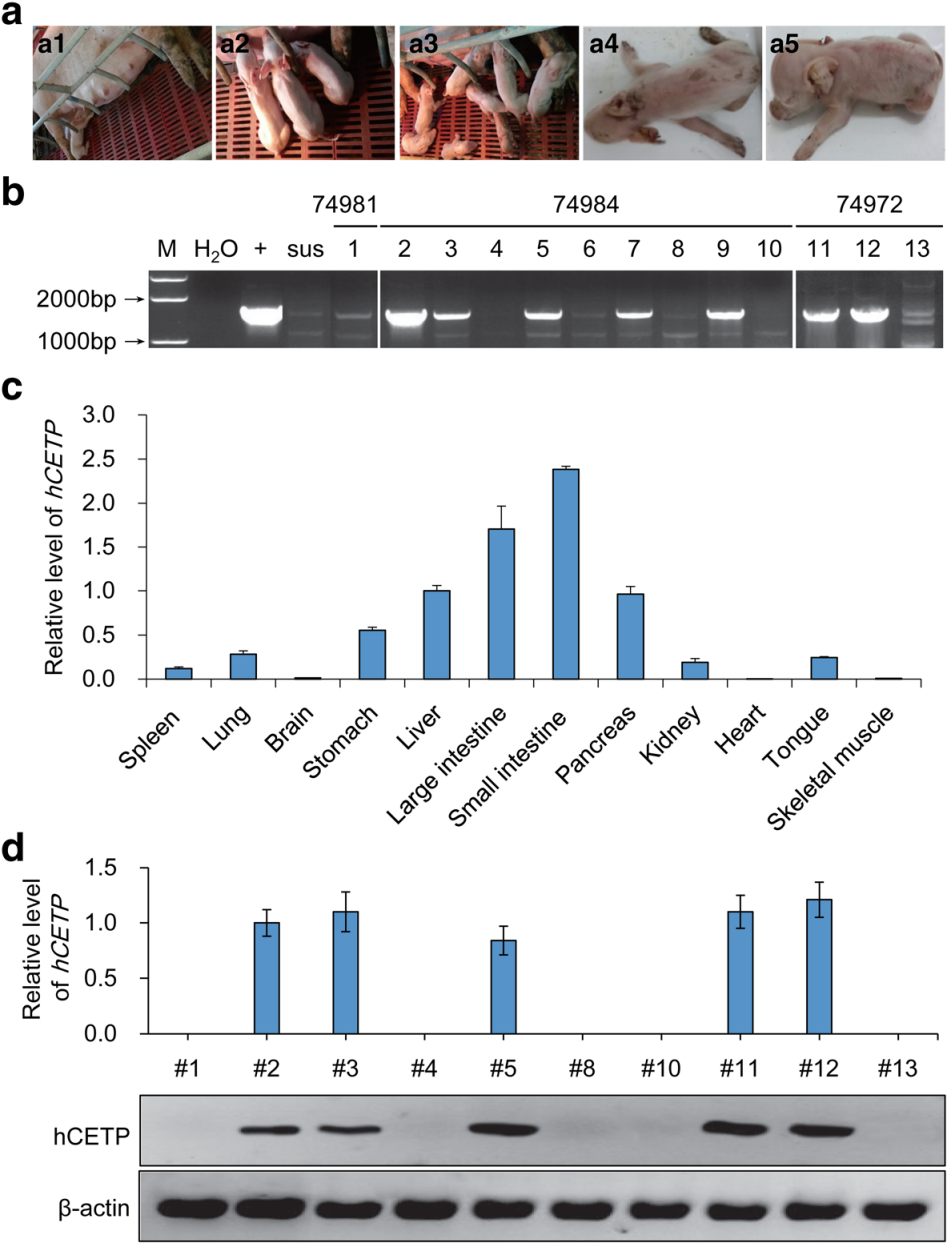
Identification of *hCETP* transgenic pigs. **a** Identification results of 13 piglets by PCR. Piglets Nos. 2, 3, 5, 7, 9, 11, 12 were *hCETP* transgenic pigs. “+” indicates that the template is the pApoC3-hCETP-IRES-EGFP plasmid; “sus” indicates that the template is DNA from wild-type pig; “H_2_O”indicates that the template is water. **b** Generation of 13 piglets. B1: Piglet No. 1 born from sow No. 74981; B2: Piglets No. 11–13 born from sow No. 74972; B3: Piglets No. 2–10 born from sow No. 74984; B4: Piglet No. 6 was maldeveloped; B5: Piglet No. 7 was maldeveloped; **c** Expression pattern of *hCETP* in tissues of piglet No. 7; **d:** Western blot identification of CETP protein in piglets No. 1, 2, 3, 4, 5, 8, 10, 11, 12, 13

We obtained a total of 3 litters of pigs from 8 embryo transfers (as shown in Fig. 1a). In one litter, one female pig (No. 1) was born alive. In another litter, 9 female piglets (from No. 2 to No. 10) were born. However, 3 of them died due to deformities or developmental defects and 2 (No. 6 and No. 7) were so weak that they were euthanized to obtain tissues to measure the expression of CETP in vivo. In the last litter, 3 female piglets (No. 11 to No. 13) were born and thrived well.

### Identification of hCETP transgenic pigs

Tissue samples of each piglet were taken and DNA was extracted, and the DNA level was identified by PCR. The results showed that pigs No. 2, 3, 5, 7, 9, 11, 12, and were positive *hCETP* transgenic pigs, and No. 1, 4, 6, 8, 10, and 13 were negative cloned pigs (as shown in Fig. 1b).

RNA was extracted from 12 tissues of piglets No. 6 and No. 7 and detected by real-time PCR. The expression of *hCETP* was not detected in the liver of piglet No. 6, while the liver of piglet No. 7 had high expression (Fig. 1c). The expression of *hCETP* in the tissues of piglet No. 7 (Fig. 1c) was consistent with theoretical levels, and *hCETP* expression in the liver and intestines of the piglets was the highest. The ApoC3 promoter successfully drove the specific expression of the target gene in the liver and intestine. Western blotting also identified that *hCETP* was expressed in the plasma of No. 2, 3, 5, 11, 12 (Fig. 1d).

### Animal characteristics

The body weights of the *hCETP* transgenic and unmodified pigs showed an upward trend with age as shown in Additional file 1: Figure S1-C. However, there was no significant difference between *hCETP* transgenic pigs and unmodified pigs at each month of age (*P* > 0.05).

**Fig. 2:**
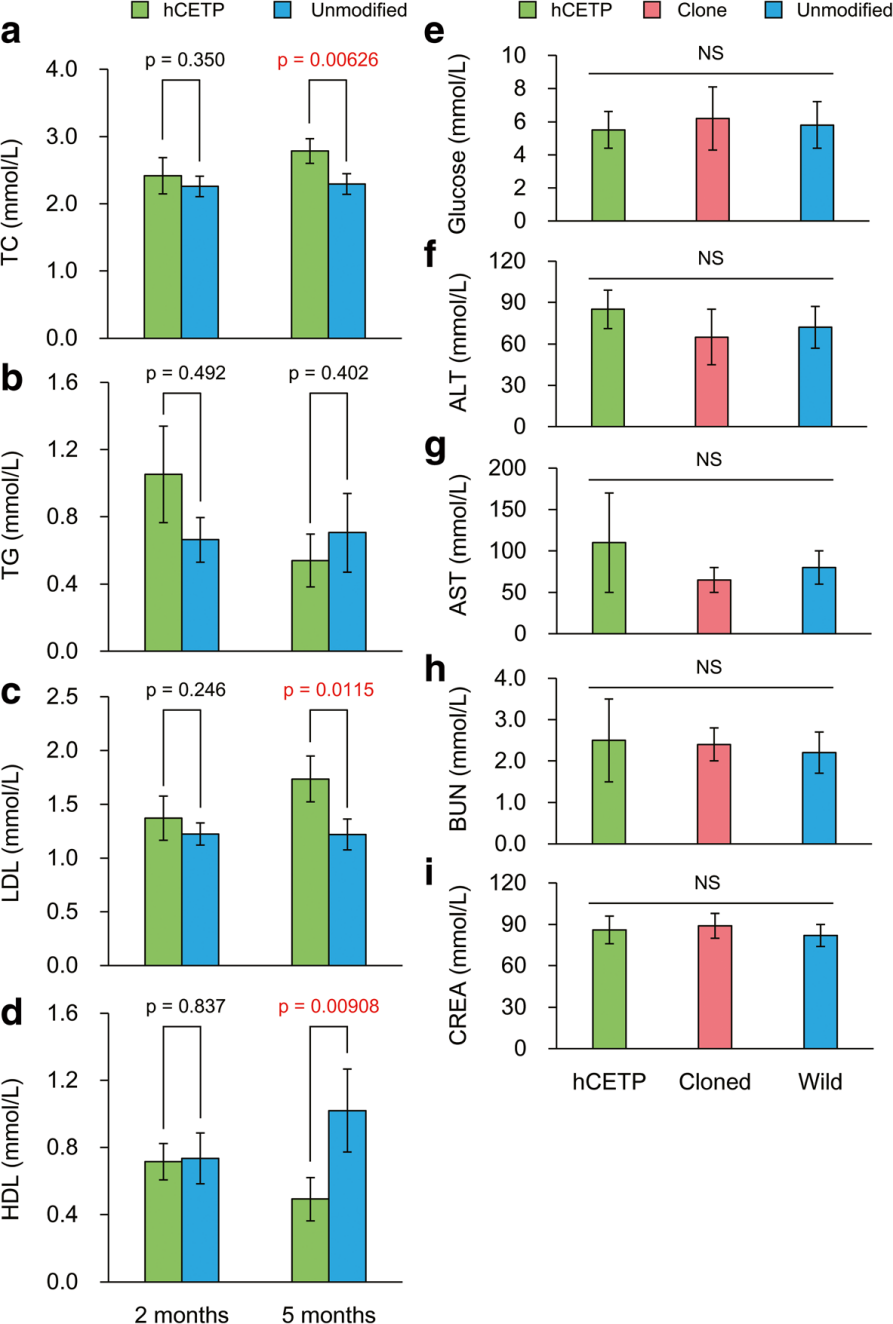
Biochemical index changes in *hCETP* transgenic pigs. **a** TC: total cholesterol; **b** TG: triglyceride; **c** LDL: low density lipoprotein; **d** HDL: high density lipoprotein; **e** Glucose level at 5 months; **f** ALT (alanine transaminase) level at 5 months; **g** AST (glutamic-oxaloacetic transaminase) level at 5 months; **h** BUN (blood urea nitrogen) level at 5 months; **i** CREA (creatinine.) at 5 months

The plasma samples from *hCETP* transgenic, cloned, and unmodified pigs were subjected to analysis. Each group contained five pigs. The lipid profile is shown in Fig. 2a-d. At 2 months of age, the lipid profile was similar between *hCETP* transgenic and unmodified pigs, which showed no significant differences in plasma TC, TG, LDL, and HDL levels (*P* > 0.05). However, TC and LDL levels were increased in *hCETP* transgenic pigs compared with unmodified pigs at 5 months (*P <* 0.05). And HDL levels were decreased in transgenic pigs (*P* < 0.05). However, there was still no significant difference between these two groups in TG levels (*P* > 0.05).

Results showed that there were no significant differences in blood glucose levels of *hCETP* transgenic, cloned, and unmodified pigs (*P* > 0.05) (Fig. 2e). Moreover, liver and renal function of *hCETP* transgenic and cloned pigs were normal (Fig. 2f-i). This indicated that the process of human gene *CETP* integration into the pig genome did not affect the metabolism of glucose or liver and renal function.

### Plasma metabolic profiling

**Fig. 3:**
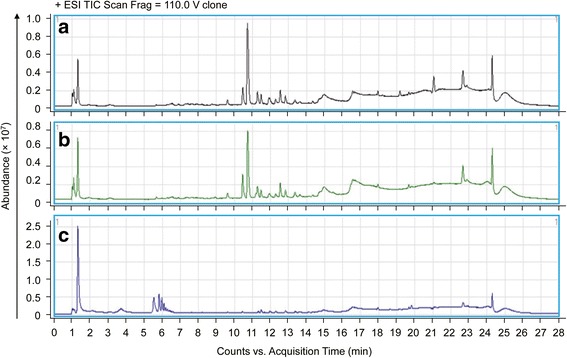
Typical RRLC-QTOF/MS chromatograms of pig plasma samples acquired in positive mode. **a** cloned pig; **b** *hCETP* transgenic pig; **c** unmodified pig

The RRLC-QTOF/MS chromatograms for *hCETP* transgenic, cloned, and unmodified pigs are shown in Fig. 3 and Additional file 1: Figure S2, which indicate peak distributions in positive mode (ESI^+^) and negative mode (ESI^−^). From these two mode analyses, we found that these spectra include thousands of plasma metabolites, and there were overlapping peaks among the 3 groups. Moreover, excellent separations among these 3 groups were analyzed, and the results indicated that their plasma metabolites were significantly different from each other.

**Fig. 4:**
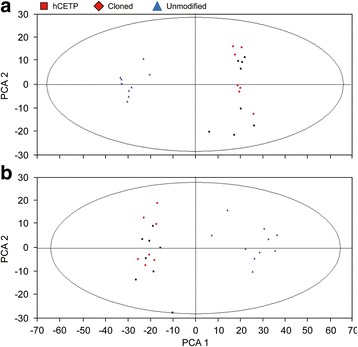
PCA scores plot in different mode. **a** PCA scores plot for the top two components discriminated based on the data in ESI^+^ mode. **b** PCA scores plot for the top two components discriminated based on the data in ESI^−^ mode. (): *hCETP* transgenic pig; (): cloned pig; (): unmodified pig

After pretreatment of the data by XCMS software, PCA analysis was used to observe the components classification trend of transgenic, cloned, and unmodified pigs in the ESI^+^ mode. The classification effect of PCA worked well and showed that there were significant differences between genetically modified pigs (including *hCETP* transgenic pigs and negative cloned pigs) and unmodified pigs. However, this plot could not classify *hCETP* transgenic pigs and cloned pigs. PCA analysis correctly displayed the different components attribution in cloned and unmodified groups (Fig. 4a). Similar to ESI^+^ mode, PCA analysis in ESI^−^ mode could also distinguish the components of cloned and unmodified pigs (Fig. 4b).

In order to observe the classification between the two groups, we used the supervised learning method PLS-DA to further analyze the data. We chose the modeling component to construct the PLS score plot (as shown in Additional file 1: Figures S3 and S4). The results showed that the PLS-DA method could clearly show the classification of CETP transgenic, cloned, and unmodified pigs. There was no overlap among the 3 groups in the PLS-DA plot, which indicated that there was a significant difference between any two groups. For evaluating the effect of the PLS-DA model, we calculated R^2^X, R^2^Y, and Q^2^. These parameters indicate the fit and prediction ability, respectively [16]. The results revealed that the PLS-DA model was valid.

## Discussion

Whether results from rodent research can be directly translated into human clinical trials is still debated. Large animal models that recapitulate human disease pathophysiology are thus attractive to researchers. The rapid development of genetic engineering technologies offers the possibility to do genetic modification in large animals. Here, we created *hCETP* transgenic pigs that overexpress CETP in a liver- and intestine-specific manner. In order to overexpress CETP specifically in liver and intestine, similar to the human expression profile, we chose the tissue-specific promoter ApoC3, which has been confirmed by Herrera et al. [6]. The present study using mass spectrometry metabolomics showed that the plasma metabolite profile of *hCETP* transgenic pigs differed from cloned pigs and unmodified pigs.

It has been revealed that CETP plays a physiological role in modulating vascular lipoproteins. Previous human genetic research and animal studies have concluded that CETP is proatherogenic, and inhibiting its activity should reduce CVD risk [17]. However, some studies of genetic *CETP* deficiency do not show a strong association with CVD risk [18, 19]. A genome-wide association study has identified 6 new loci associated with CVD, which included the *CETP* gene [20]. Our study also showed that CETP should be proatherogenic, because along with the increase of age, TC and LDL levels of *hCETP* transgenic pigs were increased and significantly higher than those of normal pigs. However, changes of blood lipid levels in *hCETP* transgenic pigs were not as significant as those of CETP transgenic mice or rats [3, 6]. One reason for this result may be the low copy number of foreign *CETP* genes integrating into the genomes of pigs due to using a plasmid vector by liposome transfection [21]. Another is that all generated *hCETP* transgenic pigs were heterozygotes produced by somatic cell nuclear transfer [22]. If homozygotes of *hCETP* transgenic pigs were generated after mating with each other, *CETP* gene expression could be enhanced. Pigs can develop AS under natural conditions, but require longer cycles. While high-fat and high-cholesterol diet feeds can accelerate AS formation, the higher economic costs limit the application of this method [23, 24]. In the last few decades, rabbits have been the main model of AS research for their extreme sensitivity to cholesterol, with CETP being highly expressed and AS plaques forming in a short time [25]. The greatest potential advances of genetically modified pigs in cardiovascular disease studies are that they could mimic human disorders and elucidate disease etiology. The heart, coronary vasculature, and blood flow of pigs is very similar to those of human beings, and overall, pigs can be used for drug, stent, or related interventional research, which can be detected by intracoronary imaging such as intravascular ultrasound (IVUS) or optical coherence tomography (OCT) [26–29]. The successful generation of *hCETP* transgenic pigs has laid the foundation for an excellent model to decipher the mechanisms of initiation and progression of AS.

The metabolomics of transgenic pigs was analyzed by liquid phase mass spectrometry. After two methods of analysis, the results showed that *hCETP* transgenic positive, cloned, and unmodified pigs showed significant differences between the three metabolic components. First, a comparison of biochemical criteria in the three groups showed that *hCETP* transgenic pigs are different from cloned and control pigs, and there were no significant differences between cloned and unmodified pigs. However, the metabolic components between cloned pigs and unmodified pigs can be distinguished by PCA analysis, indicating that the metabolic components of the two are not identical. Somatic cell nuclear transfer technology requires that single somatic cell nuclei are introduced into enucleated oocytes containing some free DNA, which may have contributed to the variation. In addition, epigenetic modifications, intestinal bacteria, and intrauterine environmental factors may have an impact on the metabolic components of cloned pigs. Studies have also shown clear differences in phenotypic or metabolic components between cloned and normal animals [30–32].

The underlying mechanism by which CETP transfers lipoprotein is still not clearly understood, and the fundamental function of CETP in nature remains unknown [9, 33]. The PLS-DA model not only distinguished the metabolic components of genetically modified pigs from unmodified pigs, but also showed discrepancy between transgenic positive and cloned pigs. The different metabolic components between *hCETP* transgenic and unmodified pigs might be closely associated with the physiological function of CETP, having effects on lipid metabolism, lipoprotein oxidation, inflammation, and fat synthesis [34]. Establishing the different metabolic components of *hCETP* transgenic positive, cloned, and unmodified pigs also needs further validation to identify the specific components, and then they can be analyzed in regards to possible mechanisms and related functions. The limitation of present study was the small sample size with only 5 pigs in each group, and a larger sample size would be more indicative of variation between animals in the multivariate analysis.

## Conclusions

In conclusion, this report describes a model of lipid metabolic disturbance created by transgenesis in a large animal. The *hCETP* transgenic pigs showed hypercholesterolemia, and metabolomic analysis found CETP-related metabolic components. Therefore, this model should be valuable for further research into the mechanisms of CETP in the development of atherosclerosis.

## Additional file

Additional file 1: Figure S1. Eukaryotic expression vector construct and its expression in pig fibroblast cells. (A) Map of the 7728 bp plasmid. PApoC3: promotor specific expression in liver and intestine; *hCETP*: human *CETP* gene. IRES: internal ribosome entry sites; EGFP: enhanced green fluorescent protein. (B) Expression of pApoC3-hCETP-IRES-EGFP plasmid in pig fibroblast cells. “+”: positive control; “S”: transfected by plasmid of pig fibroblast cells; “-”:negative control; “H_2_O”: H_2_O. C: Body weights of *hCETP* transgenic pigs. **Figure S2.** Typical RRLC-QTOF/MS chromatograms of pig plasma samples acquired in negative mode. (A) cloned pig; (B) *hCETP* transgenic pig; (C) unmodified pig. **Figure S3.** PLS-DA scores plot for components discriminated based on the data in positive mode. (A) transgenic pig and unmodified pig; (B) cloned and unmodified pig; (C) transgenic and cloned pig. (red square): positive pig; (red diamond): cloned pig; (blue triangle): unmodified pig. **Figure S4.** PLS-DA scores plot for components discriminated based on the data in negative mode. (A) transgenic pig and unmodfied pig; (B) cloned and unmodified pig; (C) transgenic and cloned pig. (red square): positive pig; (red diamond): cloned pig; (blue triangle): unmodified pig. (DOCX 1061 kb)

## Abbreviations

AS: Atherosclerosis
CE: Cholesteryl esters
CETP: Cholesteryl ester transfer protein
CVD: Cardio-vascular disease
HDL: High-density lipoprotein
IVUS: Intravascular ultrasound
LDL: Low-density lipoprotein
OCT: Optical coherence tomography
TC: Total cholesterol
TG: Triglycerides
