# Supplementary material for: A novel swine model for evaluation of dyslipidemia and atherosclerosis induced by human CETP overexpression
Source: Lipids Health Dis. 2017 Sep 11;16:169. doi: 10.1186/s12944-017-0563-x (PMC5594531; doi:10.1186/s12944-017-0563-x)
Supplement: Additional file 1: Figure S1. — Eukaryotic expression vector construct and its expression in pig fibroblast cells. (A) Map of the 7728 bp plasmid. PApoC3: promotor specific expression in liver and intestine; hCETP: human CETP gene. IRES: internal ribosome entry sites; EGFP: enhanced green fluorescent protein. (B) Expression of pApoC3-hCETP-IRES-EGFP plasmid in pig fibroblast cells. “+”: positive control; “S”: transfected by plasmid of pig fibroblast cells; “-”:negative control; “H2O”: H2O. C: Body weights of hCETP transgenic pigs. Figure S2. Typical RRLC-QTOF/MS chromatograms of pig plasma samples acquired in negative mode. (A) cloned pig; (B) hCETP transgenic pig; (C) unmodified pig. Figure S3. PLS-DA scores plot for components discriminated based on the data in positive mode. (A) transgenic pig and unmodified pig; (B) cloned and unmodified pig; (C) transgenic and cloned pig. (red square): positive pig; (red diamond): cloned pig; (blue triangle): unmodified pig. Figure S4. PLS-DA scores plot for components discriminated based on the data in negative mode. (A) transgenic pig and unmodfied pig; (B) cloned and unmodified pig; (C) transgenic and cloned pig. (red square): positive pig; (red diamond): cloned pig; (blue triangle): unmodified pig. (DOCX 1061 kb) [file 12944_2017_563_MOESM1_ESM.docx]

**A novel swine model for evaluation of dyslipidemia and atherosclerosis induced by human *CETP* overexpression**

**Additional file 1:**

**Figure S1.** Eukaryotic expression vector construct and its expression in pig fibroblast cells. **(A)** Map of the 7728 bp plasmid. PApoC3: promotor specific expression in liver and intestine; *hCETP*: human *CETP* gene. IRES: internal ribosome entry sites; EGFP: enhanced green fluorescent protein. **(B)** Expression of pApoC3-hCETP-IRES-EGFP plasmid in pig fibroblast cells. “+”: positive control; “S”: transfected by plasmid of pig fibroblast cells; “-”:negative control; “H_2_O”: H_2_O. **C:** Body weights of *hCETP* transgenic pigs.

**Figure S2.** Typical RRLC-QTOF/MS chromatograms of pig plasma samples acquired in negative mode. **(A)** cloned pig; **(B)** *hCETP* transgenic pig; **(C)** unmodified pig.

**Figure S3.** PLS-DA scores plot for components discriminated based on the data in positive mode. **(A)** transgenic pig and unmodified pig; **(B)** cloned and unmodified pig; **(C)** transgenic and cloned pig. (): positive pig; ( ): cloned pig; (): unmodified pig.

**Figure S4.** PLS-DA scores plot for components discriminated based on the data in negative mode. **(A)** transgenic pig and unmodfied pig; **(B)** cloned and unmodified pig; **(C)** transgenic and cloned pig. (): positive pig; ( ): cloned pig; (): unmodified pig.


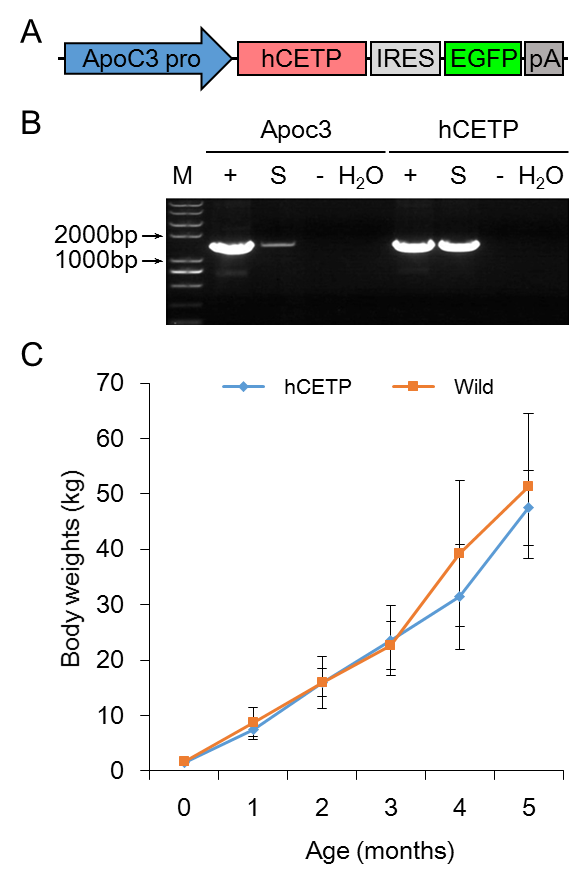


**Figure S1**


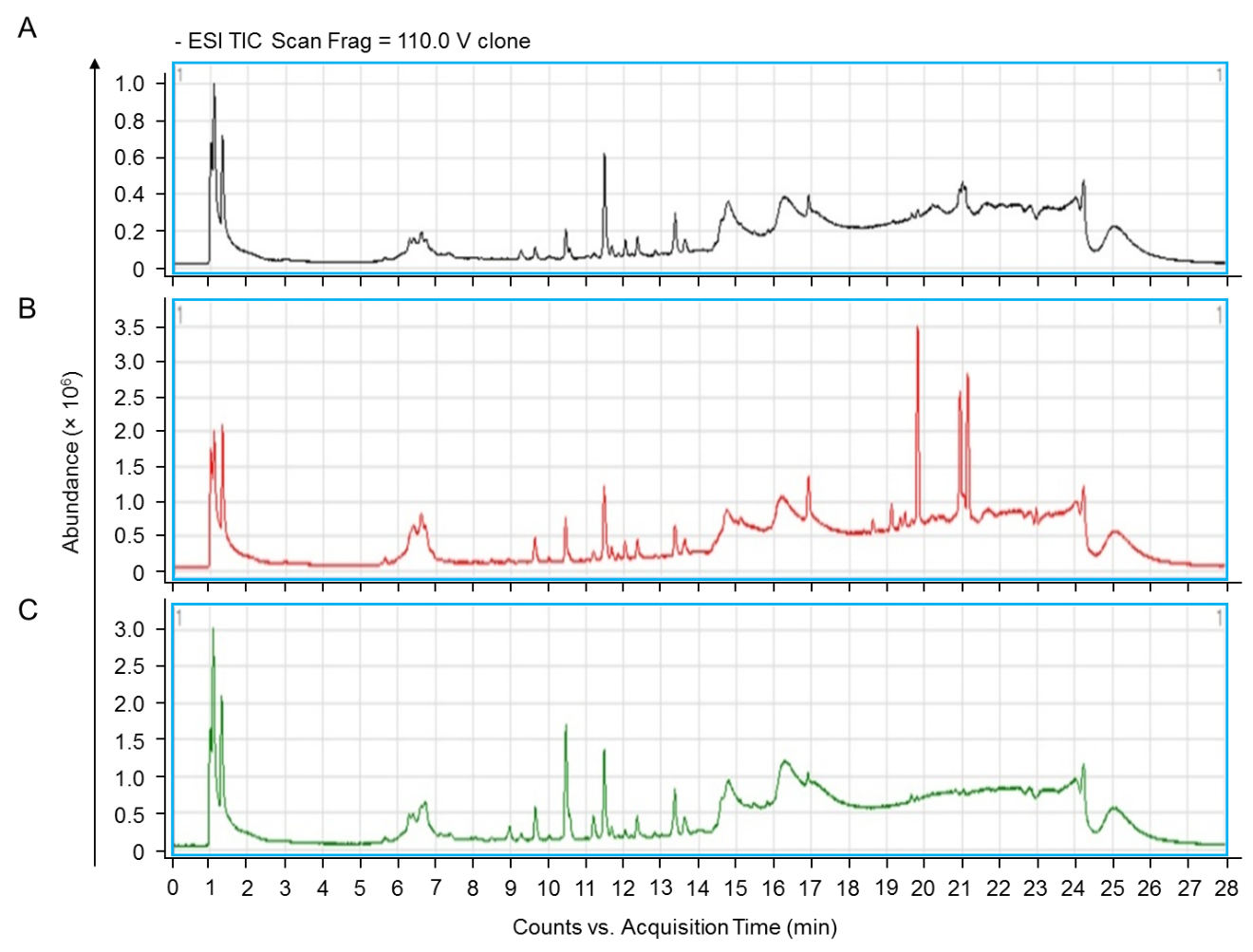


**Figure S2**

**
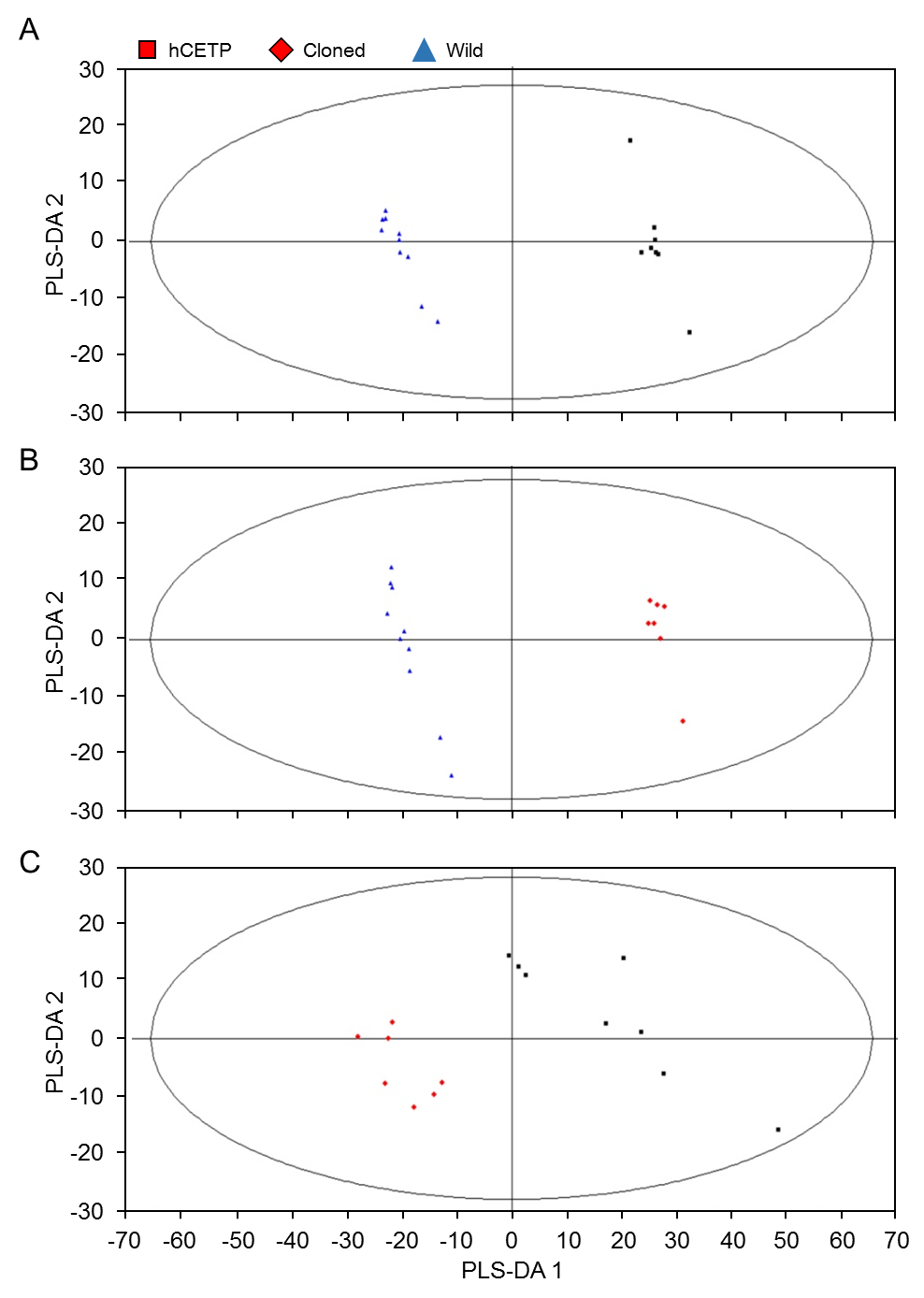
**

**Figure S3**


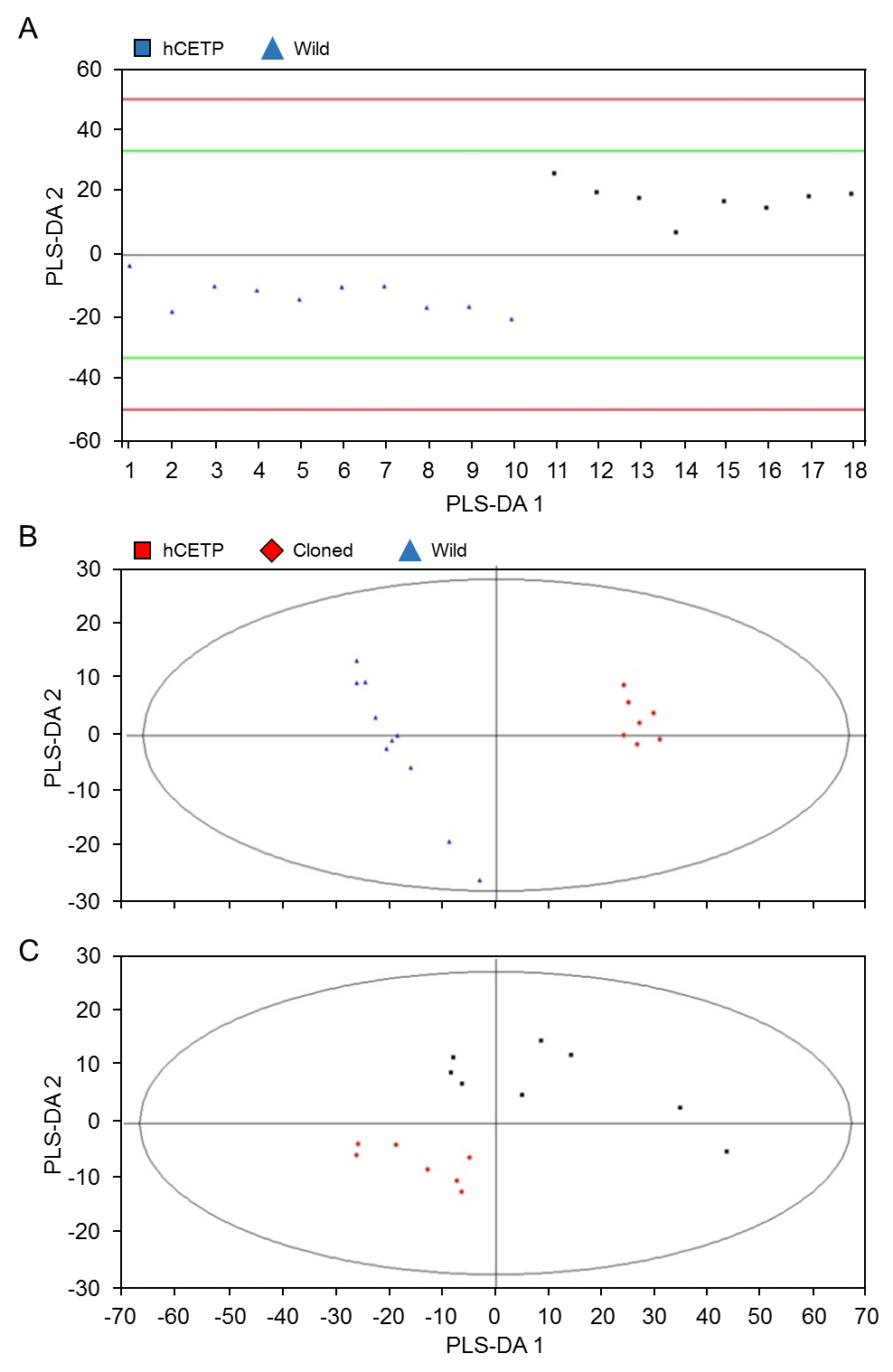


**Figure S4**
